# A case of primary retroperitoneal amyloidoma resected laparoscopically

**DOI:** 10.1016/j.eucr.2021.101711

**Published:** 2021-05-13

**Authors:** Yoshikazu Kuroki, Kaoru Kimura, Koji Harimoto, Keiichiro Nishikawa, Naoki Hosaka, Junji Uchida

**Affiliations:** a Department of Urology, Osaka City University Graduate School of Medicine, 1-4-3 Asahi-machi, Abeno-ku, Osaka 545-8585, Japan; b Department of Urology, Fuchu Hospital, 1-10-17, Hiko-Town, Izumi-City, Osaka, 594-0076, Japan; c Department of Pathology, Fuchu Hospital, Japan

**Keywords:** Amyloidoma, Amyloidosis, Laparoscopic surgery, Retroperitoneal tumor

## Abstract

Amyloidosis is known as a group of diseases that causes various disorders because of deposition of amyloid protein in various organs. Amyloidosis occurring in the retroperitoneum is a rare disease. We report a 75-year-old male patient presented to our hospital because he was identified with a retroperitoneal mass incidentally by CT. Laparoscopic surgery was performed to resect the tumor. In the histopathological specimen, amyloid was found in the fibrous soft tissue by Congo red staining. This is the first report to document a primary solitary amyloidosis of the retroperitoneum without systemic amyloidosis, which was resected using the laparoscopic approach.

## Introduction

Amyloidoma constitutes a tumor-like deposit of amyloid in various organs or tissues with no evidence of systemic amyloidosis. Some cases of primary amyloidomas which occurred in the brain, cutaneous tissue, and soft tissue of the extremities were surgically resected and their prognoses were relatively good.^1^ However, there are few reports regarding amyloidoma occurring in the retroperitoneal space. To our knowledge, there is no surgically-resected case of retroperitoneal amyloidoma.

Our purpose is to describe the case of a 75-year-old man who underwent laparoscopic surgery of the retroperitoneal amyloidoma, and to discuss the clinical presentation and outcomes with the help of previous reported cases in the literature.

## Case presentation

A 75-year-old male patient presented to our hospital because he was identified with a retroperitoneal mass incidentally by CT, which had been performed to investigate the cause of abdominal numbness in the left side.

**Fig. 1:**
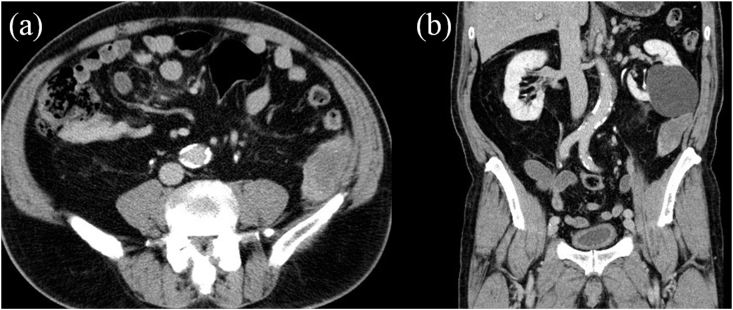
Abdominal CT images (a: axial, b: coronal view) showed a 7-cm-diameter spindle shaped lesion just inferior to the left renal cyst. The mass showed a slight contrast effect.

**Fig. 2:**
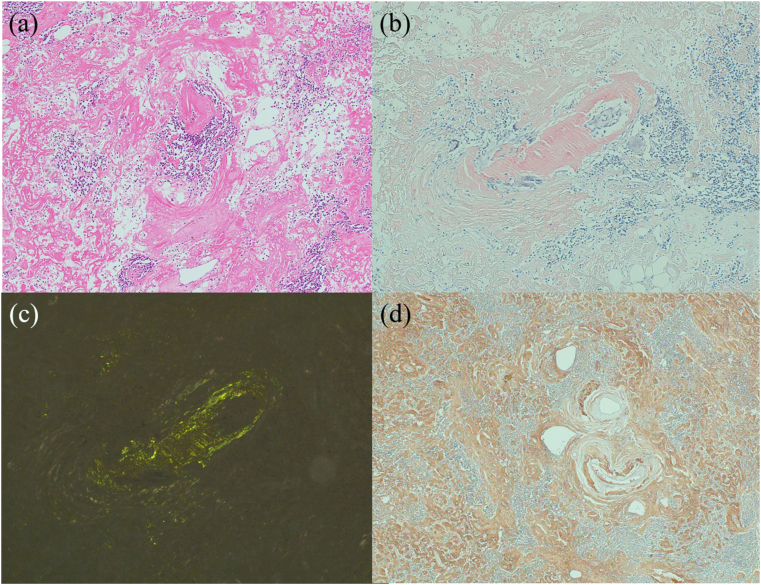
(a) Haematoxylin and eosin-stained histologic sections showed deposits of eosinophilic, amorphous material. (b) Congo red-stained section showed amyloid deposition. (c) Congo red-stained section under a polarizing microscope showed an apple-green birefringence. (d) Immunohistological staining showed positivity for kappa light chain. (For interpretation of the references to colour in this figure legend, the reader is referred to the Web version of this article.)

He had bilateral cataract surgery and cervical laminectomy in the past. There was nothing remarkable in his family history. Physical examination showed normal vital signs. Pulmonary, cardiac, and rectal examinations revealed no abnormal findings. Laboratory findings including tumor markers such as CEA, CA19-9, and AFP and urinalysis were within normal range. Electrocardiography and echocardiography revealed no abnormalities. Abdominal CT showed a 7-cm-diameter spindle-shaped lesion with calcification just inferior to the left renal cyst. The mass showed a slight contrast effect (Fig. 1). MRI showed a 6 × 5 × 3 cm, partly irregular, well-defined lesion, the intensity of which was comparable with the skeletal muscle. Gadolinium enhancement was intense at the peripheral part. At this point, the differential diagnosis of the retroperitoneal tumor was leiomyoma, leiomyosarcoma, or schwannoma. Therefore, laparoscopic surgery was performed. The intraoperative finding showed the tumor adhered to Gerota's fascia, lateroconal fascia, and peritoneum. The histopathological specimen showed that its margin was clear and the tumor was fibrous soft tissue containing vessels, nerves, and fat. The connective tissue portion showed deposits of amorphous eosinophilic material and marked hyalinization with deposits of plasma cells (Fig. 2-a). Congo red staining was positive in the connective tissue, indicating amyloid deposition (Fig. 2-b,c). Immunohistological staining was positive for kappa light chains (Fig. 2-d).

The patient was subsequently investigated for underlying diseases. Urinalysis was negative for Bence-Jones protein. M-gradient was not observed in the serum electrophoresis, and serum beta-2 microgloblin and immunoglobulin were within normal range. Serum kappa free light chain(19 mg/dL), lambda free light chain(22.9 mg/dL), and a kappa lambda free light chain ratio(0.82) were normal. There was no evidence of systemic amyloidosis, plasmacytoma, or multiple myeloma. Therefore, we diagnosed the patient with a primary localized amyloidoma. The patient underwent no further treatment. He has remained asymptomatic, and follow-up CTs have revealed no recurrent amyloidoma for 4 years.

## Discussion

Amyloidoma is termed as a tumor-like deposit of amyloid in various organs or tissues without systemic amyloidosis.^2^ Our report demonstrated a case of primary solitary amyloidoma in the retroperitoneum. To our best of knowledge, this may be the first demonstration regarding surgical treatment of amyloidoma in the retroperitoneum using the laparoscopic approach with no recurrence.

Nine cases of retroperitoneal amyloidosis have been previously reported.^3^^,^^4^ The data available have shown that these cases were secondary amyloidosis associated with malignancies such as multiple myeloma, renal cancer, and plasmacytoma. Therefore, many cases were treated by chemotherapy or radiation therapy and were diagnosed by biopsy specimens. In almost all cases, CT images showed a diffuse and irregular mass. On the other hand, the images of our case revealed a solitary mass with a clear margin. Moreover, there have been no cases of surgical resection reported. Retroperitoneal amyloidosis is usually asymptomatic and difficult to detect in the early stage, but our case was detected incidentally by a CT scan. Moreover, our case had no potential underlying cause such as multiple myeloma or macrogloblinemia. There was a possibility that these factors contributed to its complete surgical resection.

Historically, amyloidosis was classified as primary and secondary or systemic and localized. Several amyloid proteins may be associated with both systemic and localized forms. The two major amyloid proteins are the AL and the AA. In our case, the amyloid type was determined immunohistochemically as AL kappa. Once the AL amyloidosis is diagnosed, underlying diseases such as multiple myeloma should be detected and treated. Our case was considered primary AL amyloidosis in the retroperitoneum, because there was no such systemic disease. Primary solitary amyloidosis has prognoses better than other types of amyloidosis.^5^ Our case also had no cause of amyloidosis and showed a solitary mass, which was laparoscopically resected, therefore we expected a good prognosis. At the present time, the patient remains asymptomatic, and the follow-up CTs have revealed no recurrent amyloidoma for 4 years.

## Conclusion

We experienced a case of amyloidoma in the retroperitoneum. We should be aware of the possibility that amyloidoma can occur in the retroperitoneum, and it may be possible to perform curative laparoscopic surgical operation for this tumor.

## Declaration of competing interest

None declared.

## Abbreviations:

AA =: amyloid A
AFP =: alpha fetoprotein
AL =: amyloid light chain
CA19-9 =: carbohydrate antigen 19-9
CEA =: carcinoembryonic antigen
CT =: computed tomography
MRI =: magnetic resonance imaging

## References

[1] Hashmi H., Dhanoa J., Manapuram S. (2018). Trigeminal amyloidoma: case report and review of literature. Cureus.

[2] Yin H., Alhasan N., Ciervo A., Zinterhofer L. (2002). Soft tissue amyloidoma with features of plasmacytoma:A case report and review. Arch Pathol Lab Med.

[3] Yokota K., Kishida D., Kayano H. (2016). A case of abdominal aortic retroperitoneal and mesenteric amyloid light chain amyloidoma. Case Rep Rheumatol.

[4] Franco-Palacios D., Tama M., Samaddar S., Yang J. (2013). Retroperitoneal amyloidosis as the presenting manifestation of Waldenstrom's macroglobulinaemia. BMJ Case Rep.

[5] Iplikcioglu A.C., Bek S., Gokduman C.A., Cosar M., Sav A. (2007). Primary solitary cervical amyloidosis: case report and review of the literature. Spine.

